# Electrospinning of Hyaluronan Using Polymer Coelectrospinning and Intermediate Solvent

**DOI:** 10.3390/polym11091517

**Published:** 2019-09-18

**Authors:** Lenka Vítková, Lenka Musilová, Eva Achbergerová, Antonín Minařík, Petr Smolka, Erik Wrzecionko, Aleš Mráček

**Affiliations:** 1 Department of Physics and Materials Engineering, Faculty of Technology, Thomas Bata University in Zlín, Vavrečkova 275, 760 01 Zlín, Czech Republic; l_davidova@utb.cz (L.V.); lmusilova@utb.cz (L.M.); minarik@utb.cz (A.M.); smolka@utb.cz (P.S.); wrzecionko@utb.cz (E.W.); 2 Center of Polymer Systems, Thomas Bata University in Zlín, tř. Tomáše Bati 5678, 760 01 Zlín, Czech Republic; 3 CEBIA-Tech, Faculty of Applied Informatics, Thomas Bata University in Zlín, Nad Stráněmi 4511, 760 05 Zlín, Czech Republic; achbergerova@utb.cz

**Keywords:** electrospinning, hyaluronan, poly(vinyl alcohol), polyethylene oxide, nanofibers, intermediate solvent, fluorescence confocal microscopy

## Abstract

In the current study, we present methods of sodium hyaluronate, also denoted as hyaluronan (HA), nanofiber fabrication using a direct-current (DC) electric field. HA was spun in combination with poly(vinyl alcohol) (PVA) and polyethylene oxide (PEO) and as a pure polymer. Nonaggressive solvents were used due to the possible use of the fibers in life sciences. The influences of polymer concentration, average molecular weight ($M_{w}$), viscosity, and solution surface tension were analyzed. HA and PVA were fluorescent-labeled in order to examine the electrospun structures using fluorescence confocal microscopy. In this study, two intermediate solvent mixtures that facilitate HA electrospinning were found. In the case of polymer co-electrospinning, the effect of the surfactant content on the HA/PVA electrospinning process, and the effect of HA $M_{w}$ on HA/PEO nanofiber morphology, were examined, respectively.

## 1. Introduction

Electrospinning, nowadays a well-established fiber-fabrication method first described in 1902 by Cooley and Morton [1,2], is based on several electrohydrodynamic phenomena [3]. The method utilizes Taylor cones, the product of electric field-induced instabilities in liquid bodies stabilized by capillary forces. However, if electric forces overcome capillary forces, a liquid jet is ejected, which is then subjected to elongation at high rates, causing a decrease in diameter up to micron fractions. Due to the large specific surface of the polymer jet, rapid evaporation of the solvent occurs, leading to the solidification of the polymer jet in the form of a nanofiber. Several instabilities may occur and disrupt the electrospinning process, causing particle formation (so-called electrospraying), bead-on-string structure formation, or branching. The preliminary cause of these phenomena is Rayleigh instability, which is surface tension-driven and electrostatically hindered. Similarly to Taylor cone formation prior to electrospinning, cone-shaped undulations may be formed on the cylindrical jet, leading to jet collapse if the charge per unit area is small, providing electrospraying or bead-on-string structured fibers, or undulation stabilization and elongation, giving branched fibers [3,4]. This technique inherently gives nonwoven mats composed of infinite fibers. In practice, DC electric fields of up to tens of kilovolts magnitude are used in most cases.

Nanofibrous materials have uses in many industry fields, for example, in porous materials [5,6,7], the fuel cell industry [8,9], petroleum engineering [10,11], and particularly biomedical applications such as wound dressing [12,13], drug delivery, [14,15], or tissue engineering [16,17]. The most popular synthetic polymers for electrospinning include polyethylene oxide (PEO), poly(lactide), and polyethyleneimine [4,18,19]. Regarding natural polymers, proteins, such as silk fibroin [20], or polysaccharides, such as alginate, cellulose, or chitosan, can be used [14]. The presence of a nanostructure was proven to enhance cell proliferation [21], which is why combined 3D printing and electrospinning techniques are widely studied for potential uses in tissue engineering (see, e.g., Mori et al. (2018) [22]).

Hyaluronan (HA) is a polysaccharide abundant in the extracellular matrix of living organisms. Its primary structure is linear, and its secondary structure is typically a twisted ribbon. Due to a rather stiff backbone chain caused by the disaccharide structure, internal hydrogen bonds, and interactions with solvents, the ternary structure is an expanded random coil [23,24]. It was experimentally proven that the presence of ions can influence coil diameter [25]. The coil structure is capable of absorbing approximately 1000 times its weight of water [26]. This provides solutions of HA with extraordinarily high viscosity at low concentrations, as well as shear-thinning behavior. Its excellent biocompatibility and solubility in water makes it a popular choice in biomedical applications [27]. HA melt processing is impossible due to its instability at high temperatures [28].

The main complications in HA solution electrospinning are the high surface tension of HA aqueous solutions, extremely high viscosity at low concentrations, preventing the formation of highly concentrated solutions, and the low evaporation rate of water. Attempts to fabricate HA nanofibers using electrospinning have been made by many researchers. A common approach to electrospinning of polymers with low spinnability is the use of a highly spinnable polymer that then serves as a dragging polymer [20,29]. Under certain conditions, this approach provides core–shell nanofibers, as was demonstrated by Ma et al. (2017) [30] using chitosan and an HA solution. To overcome the problem of high surface tension, the use of surfactants [31] or a different solvent choice is possible [27,32,33]. According to Malkin et al. (2017), a change in solvent also has a positive effect on spinnability due to introducing a polymer–solvent demixing solidification mechanism [34]. Previously, it was assumed that electrospinning is only possible above critical concentration, i.e., polymer concentration corresponding to one entanglement per chain [3]. However, using solutions of PEO and polyethylene glycol (PEG), Yu et al. (2006) demonstrated that electrospinning is possible below critical concentration [35]. It was argued that the ability to form smooth fibers via electrospinning is governed by solution elasticity [36,37,38]. On the other hand, Shenoy et al. (2005) performed electrospinning experiments on several polymer solutions, and concluded that complete stabilization of the electrospinning process is provided by a minimum of 2.5 entanglements per chain [39]. Malkin et al. (2017) argued that stabilization of the electrospinning process can be achieved at concentrations below critical if an intermediate solvent is used [34]. Experiment evidence suggests great contribution of solution elasticity, interaction parameters, surface tension, and conductivity to the electrospinning process. Ambient parameters, such as temperature and humidity, need to be taken into account as well [40]. Although there have been attempts for analysis of electrospinning jet behavior [4,41], so far none are comprehensive enough to account for all influence.

In the current study, nanofibrous mats containing HA were obtained using electrospinning. Two approaches were employed: HA co-electrospinning in a blend with highly spinnable polymers PVA and PEO, respectively, and the use of an intermediate solvent. As intermediate solvents, mixtures containing water and isopropanol (IPA), and water, ethanol EtOH, and methanol (MeOH) were used. The solvent mixtures were found with the aid of a Teas graph, incorporating the method described in Luo et al. (2010) [42]. We attempted to offer insight on the influence of shear viscosity and polymer-chain conformation in the solution on the electrospinning process. Furthermore, HA and PVA were fluorescent-labeled, which allowed the products to be observed by fluorescence confocal microscopy.

## 2. Materials and Methods

### 2.1. Materials and Chemicals

HA of $M_{w}$ 243 kDa, 370 kDa, 600 kDa, and 1180 kDa was purchased from Contipro a.s. Demineralized (DEMI) water was prepared using the Milipore Direct-Q 3UV system. PVA of $M_{w}$ 89–98 kDa, 99+% hydrolyzed, PEO of $M_{w}$ 300 and 600 kDa, respectively, EtOH absolute Spectranal, IPA puriss p.a., ACS reagent, disodium hydrogen phosphate dodecahydrate ≥99%, 4-acetamido-TEMPO free radical, 97%, dimethyl sulfoxide (DMSO) ACS reagent, ≥99,9%, Nile Blue A, dye content ≥75%, NaBH_3_CN reagent grade, 95%, pyridine anhydrous 99.8%, dibutiltin dilaurate, 95%, fluorescein isothyocyanite isomer (FITC), ≥90% and benzethonium chloride (BEC), ≥97% were purchased from Sigma Aldrich. MeOH p.a. was purchased from Lach:Ner. Sodium bromide pure was purchased from Lachema a.s. Sodium hypochlorite solution pure was purchased from Penta. NaCl PharmaGrade was purchased from SAFC. NaHCO_3_ ACS Grade was purchased from VWR.

### 2.2. HA Fluorescent Labeling

In order to prepare Nile Blue A labeled-HA (600 kDa), HA was initially oxidized according to a previously published method, Huerta-Angeles et al. (2012) [43], followed by fluorescent labeling described in Šmejkalová et al. (2017) [44].

Initially, HA (1 g) was dissolved in 10 mL of DEMI water. To the HA solution, sodium bromide (0.129 g) and disodium hydrogen phosphate (0.771 g) were added. The reaction mixture was cooled to 5 °C, followed by the addition of 4-acetamido-TEMPO (5 mg) and 450 μL of sodium hypochlorite. The reaction was carried out for 45 min under nitrogen atmosphere at 5 °C. The oxidized HA was dialyzed against DEMI water for 3 days and freeze-dried (yield: 96%). In the second step, an aqueous solution (2 wt.%) of oxidized HA (0.5 g) was stirred with Nile Blue A (92 mg) predissolved in DMSO (5 mL) for 5 h. Subsequently, NaBH_3_CN (79 mg) was added to this reaction mixture which was then stirred over night at room temperature. The crude product was precipitated by NaCl solution and IPA, and the remaining Nile Blue A was washed out using IPA. The product was then dialyzed against 0.5 wt.% NaCl and 0.5 wt.% NaHCO_3_ aqueous solutions for 2 days and against DEMI water for 3 days. The final product was obtained in a form of a blue lyophilisate (yield 78%).

### 2.3. PVA Fluorescent Labeling

PVA was labeled with FITC following the procedure published by Kaneo et al. (2005) [45]. Briefly, PVA (2.5 g) was dissolved in DMSO (66.6 mL) and pyridine (416.6 μL) under stirring at 80 °C for 24 h. FITC (83 mg) and dibutiltin dilaurate (31 μL) were added to the PVA solution and the reaction was carried out for 2 h at 95 °C in darkness. The crude product was precipitated and washed with IPA, followed by dialysis against DEMI water and lyophilization. The yield of the reaction was 88%.

### 2.4. Solutions Preparation

HA of respective $M_{w}$ was dissolved in binary and ternary solvent mixtures at 50 °C under vigorous stirring for 48 h regardless the HA $M_{w}$ and solvent mixture, to obtain completely homogenized solution. The solvent mixtures chosen for the experiments were H_2_O:IPA in 10:7 weight ratio, and H_2_O:EtOH:MeOH in 5:5:1 weight ratio.

HA/PVA blend solutions with BEC surfactant were prepared in the following way; 2 wt.% HA 600 kDa solution and 1 wt.% PVA 89–98 kDa solution were prepared separately by dissolving the respective polymers in DEMI water for 24 h at elevated temperature (50 °C for HA, and 80 °C for PVA). BEC aqueous solutions of the following concentrations: 1 wt.%, 2 wt.%, 5 wt.%, and 10 wt.% were prepared separately as well. The final solutions were prepared by mixing 2.5 g of HA solution with 2 g of PVA solution. After the components were properly mixed, 0.03 g of BEC solution of respective concentration was added and the solution was mixed properly. Slight turbidity appearance was observed upon the surfactant addition. For the purpose of confocal microscopy, 4% of the respective polymer content was replaced by fluorescent labeled analogue.

HA/PEO 2 wt.% blend solutions were prepared by mixing the polymers at 1:1 weight ratio and dissolving them in DEMI water by stirring vigorously at room temperature for 48 h. HA $M_{w}$ used were 243, 370, and 600 kDa. PEO $M_{w}$ used were 300 and 600 kDa. For the purpose of confocal microscopy, 4% of HA content was replaced by Nile Blue A labeled HA.

### 2.5. Electrospinning Equipment

**Figure 1:**
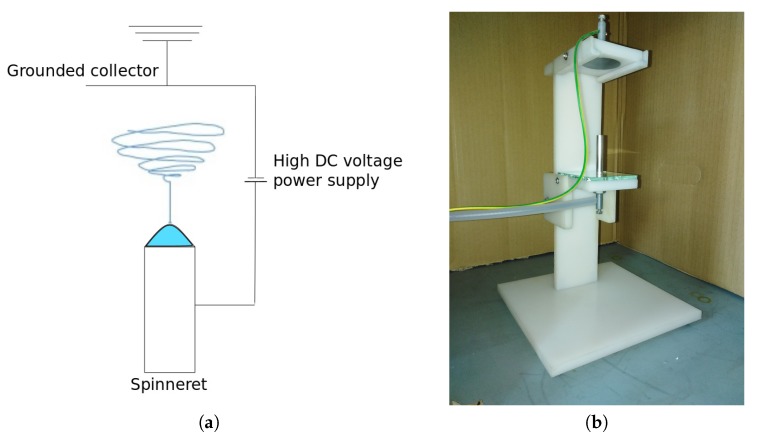
Electrospinning device: (**a**) device scheme and (**b**) device used in experiments.

A homemade electrospinner consisting of high DC voltage power supply Spellman SL150, a grounded metal collector, 40.3 mm in diameter, and a simple metal rod spinneret, 8 mm in diameter, were used in the study (see Figure 1). The tip-to-collector distance was kept at 76 mm. Experiments were conducted in air atmosphere at room temperature and humidity, and normal pressure. The fibers were collected using a recycled paper substrate to ensure good adhesion.

### 2.6. Characterization

Dynamic viscosity was determined using a Malvern Kinexus Pro+ rotational rheometer with cup-and-bob geometry. The measurements were conducted at 25 °C at 11 different shear rates ranging from 0.1 to 10 s^−1^.

Portable conductometer Mettler Toledo Seven2Go Pro was used to determine the conductivity of the solutions. Each solution was measured 3 times at room temperature.

Surface tension was determined by a pendant drop method using a Krüss Drop Shape Analyzer DSA 100. Three separate drops of each sample were measured. Each drop was measured 30 times with a 1 s delay between the measurements. Dixon’s Q-test was used to exclude the outliers. The measurement was conducted at 25 °C in an air atmosphere.

The fiber morphology analysis was done using a Phenom Pro X Scanning Electron Microscope (SEM) in the backscattered electron mode. The samples were sputtered with a layer of gold prior to the analysis. Acceleration voltage was 10 kV. Optical analysis of the images was done using ImageJ software.

An Olympus FLUOVIEW FV3000 Laser Scanning Microscope was used for fluorescence confocal microscopy. Excitation wavelenghts available were 405, 488, 561, and 640 nm. Wavelength ranges 600–640 nm and 450–520 nm were, respectively, used as emission spectra for HA labeled by Nile Blue A, and PVA labeled by FTIC. The immersion objective (*Z* = 60) with numerical aperture *A* = 1.35 was used for nanofibers observation.

## 3. Results and Discussion

The electrospinning process is highly dependent on the intrinsic properties of the spinning solution. The most prevalent were polymer $M_{w}$, concentration and polydispersity, all of which were reflected in viscosity, and also surface tension and conductivity [40]. Higher conductivity was presumed to facilitate stability in the spinning process [4], while high surface tension prevented electrospinning onset [3]. Measurement of shear viscosity was done at low shear rates in a narrow range, since the formation of a Taylor cone in sufficiently conductive fluids typically occurs without inducing high shear rates [46], and solutions are considered Newtonian liquids in this part of the process.

### 3.1. HA/PVA Blend Aqueous Solutions

In the past, electrospinning HA/PVA aqueous solutions was not possible without the addition of a small amount of surfactant. BEC was chosen due to the coil-shrinking effect on HA conformation [24], which we assumed to be beneficial in terms of electrospinning.

The surface tension of HA/PVA blend aqueous solutions was lower than the HA aqueous solutions (around 70 mN·m^−1^, see Jurošková (2017) [47]), which is likely the result of surfactant BEC addition. Surface tension decreases with increasing of BEC content. Solution conductivity was increased by the increase of BEC content as a result of BEC ionic nature (see Table 1).

HA/PVA blend aqueous solutions show the highest conductivity of the spinnable solutions used in the current study (see Table 1 and Tables 3–5). This is likely the synergic effect of dissociation of HA and PVA in water, and the addition of ionic surfactant to the solution.

**Figure 2:**
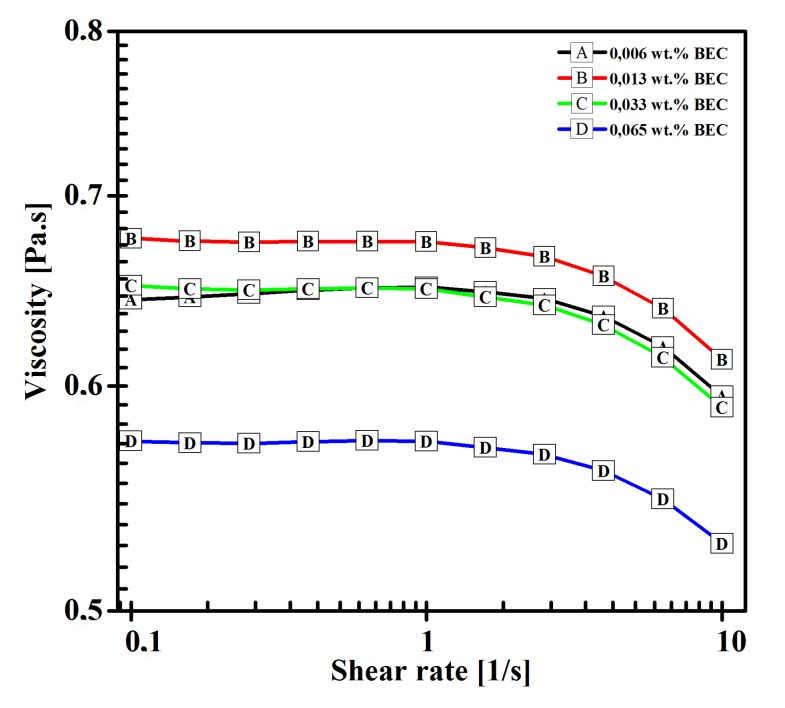
Viscosity of HA/PVA blend solutions with BEC as a function of shear rate.

Low polymer concentration causes the viscosity of the solutions to be low as well. As apparent from Figure 2, there was a quick drop of viscosity present upon addition of 0.065 wt.% of BEC.

0.065 wt.% of BEC was close to critical aggregation concentration [24] and HA coil shrinkage was expected, causing significant increase in turbidity of the solution, which was observed during the preparation, and it was in agreement with findings of Gřundělová et al. (2013) [24]. It is safe to assume that BEC effectively created an intermediate solvent to HA, and electrospinning HA is therefore encouraged not only by mixing with highly spinnable PVA, but also by the intermediate solvent effect. Due to HA precipitation, further increase of BEC content would be counterproductive.

The effect of surfactant content on electrospun-structure morphology and the electrospinning process was examined. As the BEC content increased, the minimum spinning voltage decreased (see Table 2) due to the decrease in surface tension.

**Figure 3:**
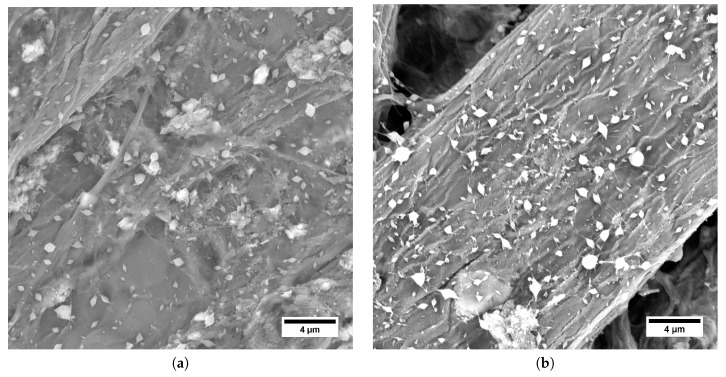
Scanning Electron Microscopy (SEM) micrographs of electrospun structures obtained from HA/PVA blend solutions with BEC. BEC content (**a**) 0.033 wt.%. and (**b**) 0.065 wt.%.

With the increase of BEC content, product morphology shifted from elongated beads to bead-on-string structured fibers (see Figure 3).

The increase in stability may be the result of increased conductivity of the solution, as argued by Reneker and Yarin (2008) [4], or the increase in polymer–polymer interactions, i.e., lowering solvent quality, which would be in agreement with the findings of Malkin et al. (2017) [34]. However, electrospinning smooth fibers was not achieved by this method. There were multiple reasons, such as insufficient solution elasticity, difference in HA and PVA viscoelastic behavior, or uneven BEC distribution, leading to formation of clumps of the respective polymers. Higher BEC content also led to the formation of multiple Taylor cones, therefore increasing the yield of the process. To proceed on this subject, it would be possible to choose a different surfactant with higher critical aggregation concentration, or alter the HA/PVA ratio in the solution in a way to increase the elongation elasticity. Both approaches would certainly lead to a better understanding of the co-electrospinning phenomenon, and might lead to smooth nanofiber production. The electrospun structures did not exceed 1 μm in diameter (see Table 2).

**Figure 4:**
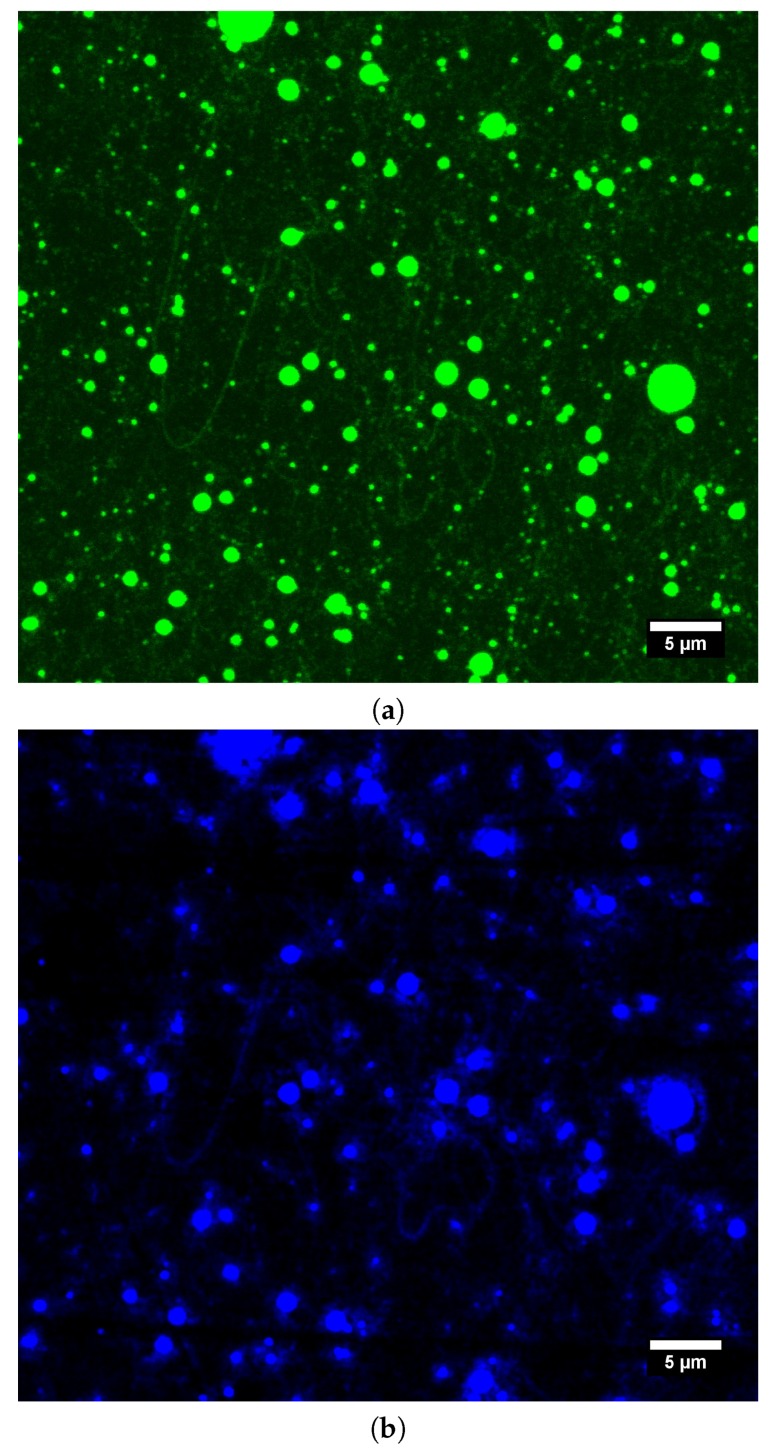

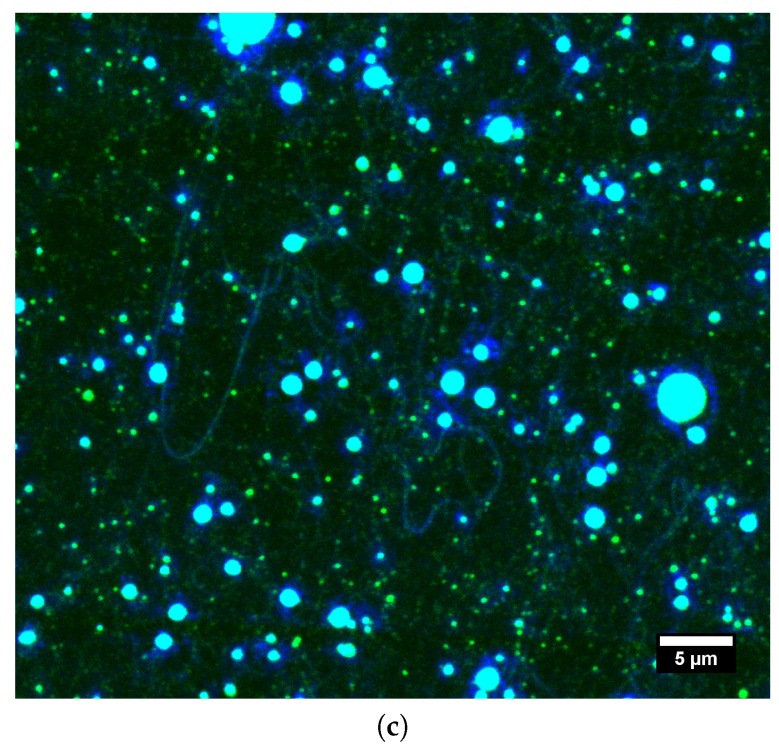
Fluorescence confocal microscope micrographs of electrospun structures obtained from Nile Blue A labeled HA 600 kDa and FITC labeled PVA 89–98 kDa aqueous solution with BEC content 0.065 wt.%. (**a**) Nile Blue A labeled HA visible. Emission spectrum 600–640 nm. (**b**) FITC labeled PVA visible. Emission spectrum 450–520 nm. (**c**) Both fluorescent labeled polymers visible—combined emission spectra.

Nile Blue A labeled HA 600 kDa and FITC labeled PVA 89–98 kDa blend aqueous solution containing 0.065 wt.% of BEC was electrospun, and the products were observed by fluorescence confocal microscopy. It is clear from Figure 4 that both polymers are present jointly in fibers, as well as beads. However, due to insufficient magnification it was not possible to determine the respective position of the polymers within the structures. The absence of some structures when illuminated by a different wavelength suggested fluctuation in the contents of the respective polymers throughout spinning.

### 3.2. HA/PEO Blend Aqueous Solutions

HA/PEO blend solutions were spun in order to produce HA containing nanofibers from aqueous solutions without use of any additional substances, such as surfactants or salts. PEO served as elasticity and shear viscosity mediator, as well as a highly spinnable polymer for co-electrospinning. In order to examine the influence of HA $M_{w}$ on the electrospinning process, PEO was used in such $M_{w}$ and concentration that would facilitate the electrospinning of all HA $M_{w}$ chosen. The PEO $M_{w}$ and concentration were found experimentally.

Surface tension of HA/PEO blend aqueous solutions was significantly higher than the one of HA in intermediate solvents solutions (see Table 3, Table 4 and Table 5) due to use of water as a solvent, but still considerably lower than HA aqueous solutions [47], caused by the surface tension-enhancing effect of HA being hindered by the presence of PEO, which induced a decrease in surface tension of the aqueous solutions instead [48].

The effect of respective polymers $M_{w}$ on the surface tension was inconclusive, as a result of the low polymer concentration used (2 wt.%). Conductivity of the solutions was significantly higher than that of the pure HA solutions (see Table 3, Table 4 and Table 5), despite the lower concentration of HA. This can be attributed to the higher dissociation of HA in water than in solvents containing alcohols. As a result of the increase in molar fraction with the decrease in $M_{w}$ while the same weight fraction of a polymer was kept, solution conductivity slightly decreased with the increase of HA $M_{w}$. As the $M_{w}$ decreased, the effect of the end groups also gained significance and, in the case of HA and PEO, contributed to conductivity as well.

**Figure 5:**
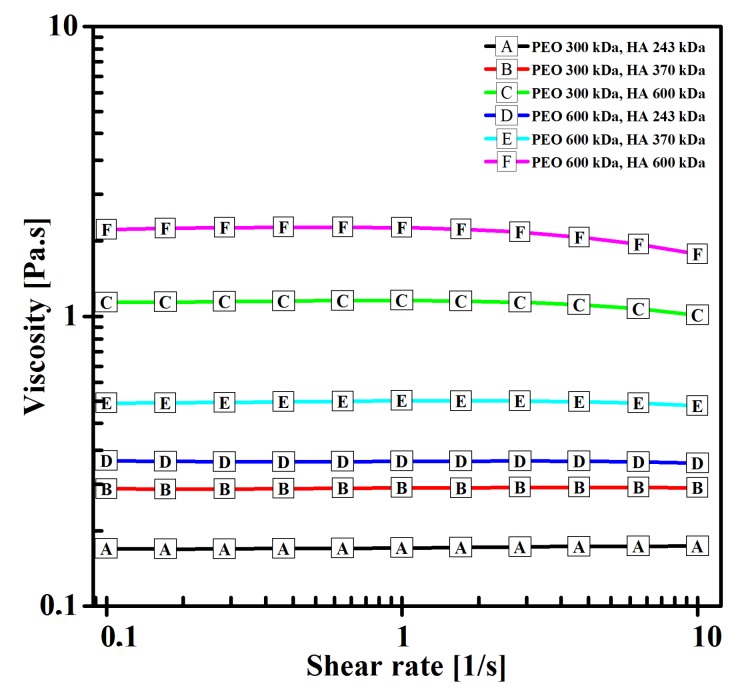
Viscosity of HA/PEO blend solutions as a function of shear rate.

Solution viscosity dropped as the $M_{w}$ of the respective polymers decreased (see Figure 5). Viscosity was significantly lower than that in the case of solutions of HA in intermediate solvents, which was one of the purposes of adding PEO into an HA aqueous solution.

In order to examine the effect of HA $M_{w}$ on the morphology of structures electrospun from HA/PEO blend aqueous solutions, the processing parameters, i.e., tip-to-collector distance and spinning voltage, were kept constant for each series of samples. All of the solutions gave bead-on-string structured fibers, which was a result of Raileygh instability acting on a conductive liquid jet in a strong electric field [4]. The structures did not exceed 1 μm in size (see Table 6).

**Figure 6:**
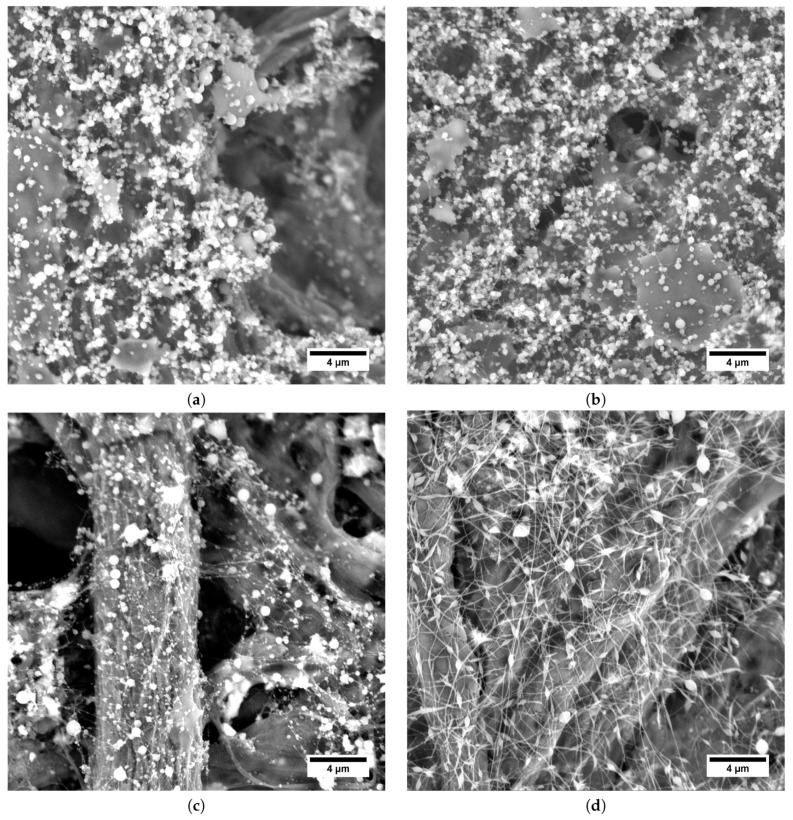

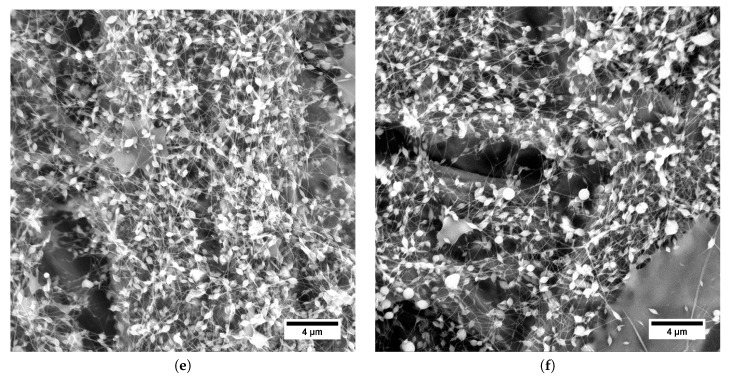
SEM micrographs of electrospun structures obtained from HA/PEO blend solutions. (**a**) HA 243 kDa, PEO 300 kDa. (**b**) HA 370 kDa, PEO 300 kDa. (**c**) HA 600 kDa, PEO 300 kDa. (**d**) HA 243 kDa, PEO 600 kDa. (**e**) HA 370 kDa, PEO 600 kDa. (**f**) HA 600 kDa, PEO 600 kDa.

As can be seen in Figure 6, the shape of the beads was influenced by the $M_{w}$ of PEO. If PEO 300 kDa (Figure 6a–c) was used, the beads were almost spherical, while for PEO 600 kDa (Figure 6d–f) strong elongation of the beads was apparent.

The significance of morphology difference could even suggest different bead origin. Another explanation is a significant shift in solution elasticity induced by the different $M_{w}$ of PEO. A great difference in viscoelasticity of the respective polymers also contributed to the uneven distribution of polymers throughout the spinning via a phenomenon known as polymer wrapping in coextrusion [49]. Further experimental examination of this phenomenon is needed in order to fully understand the causes. No effect of HA $M_{w}$ on HA/PEO fiber morphology was found in the current study, as it was likely hindered by the significant PEO content in the used samples.

**Figure 7:**
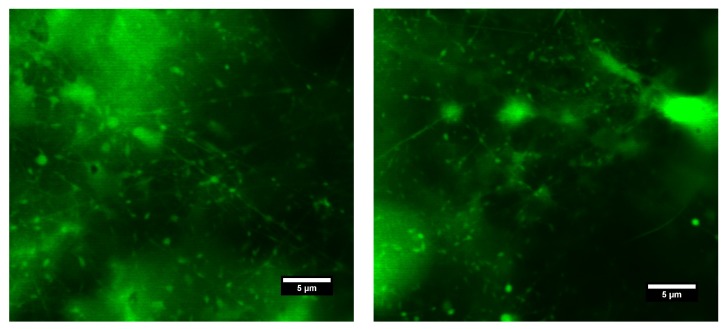
Fluorescence confocal microscope micrographs of electrospun structures obtained from the Nile Blue A Labeled HA 600 kDa and PEO 600 kDa aqueous solution. Emission spectrum: 600–640 nm.

Electrospinning of HA/PEO blend solution containing Nile Blue A labeled HA 600 kDa and PEO 600 kDa allowed us to investigate the obtained structures by using fluorescence confocal microscopy. This technique proved the presence of HA in both fibers and beads (see Figure 7). On account of PEO’s nonfluorescence, it is not visible in the figure.

### 3.3. HA Solutions in Intermediate Solvents

**Figure 8:**
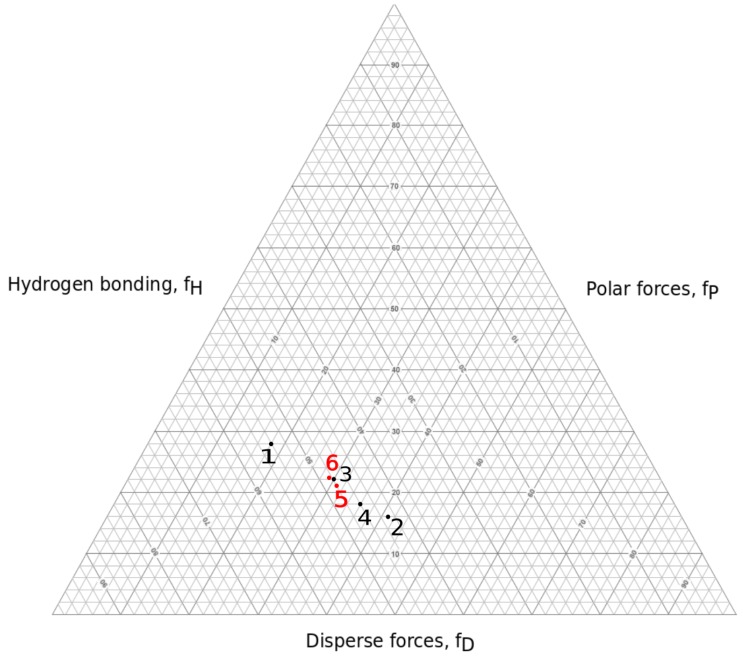
Solvent–mixture representation in Teas graph. 1: water; 2: IPA; 3: MeOH; 4: EtOH; 5: H2O:IPA 10:7; 6: H2O:EtOH:MeOH 5:5:1.

Two solvent mixture systems were chosen for the experiments: H_2_O:IPA in 10:7 weight ratio and H_2_O:EtOH:MeOH in 5:5:1 weight ratio. These were chosen with the aid of a Teas graph (see Figure 8) in such manner that they would lower the surface tension compared to water solutions, and encourage polymer–polymer interactions over polymer–solvent interactions, leading to smooth nanofiber production.

Electrospinning solutions of three different concentrations were considered for each solvent mixture and HA $M_{w}$, respectively, with the intention to find upper and lower limiting concentration for electrospinning.

The use of H_2_O:IPA an H_2_O:EtOH:MeOH mixed solvents led to a significant reduction of surface tension compared to the aqueous HA solutions, see Table 4 and Table 5 [47]. H_2_O:EtOH:MeOH solutions showed slightly higher surface tension than that of the H_2_O:IPA solutions.

No significant effect of HA concentration or $M_{w}$ on surface tension was found in the current study, which was due to very low polymer concentrations and the narrow range of concentrations used. As a consequence of HA’s ionic nature, solution conductivity was decreased as the concentration of the polymer was decreased. Electrospinning at very low concentration could therefore be hindered by two mechanisms—insufficient polymer chain entanglement, caused by low polymer concentration, and instability of the cylindrical jet, due to a decrease in conductivity.

**Figure 9:**
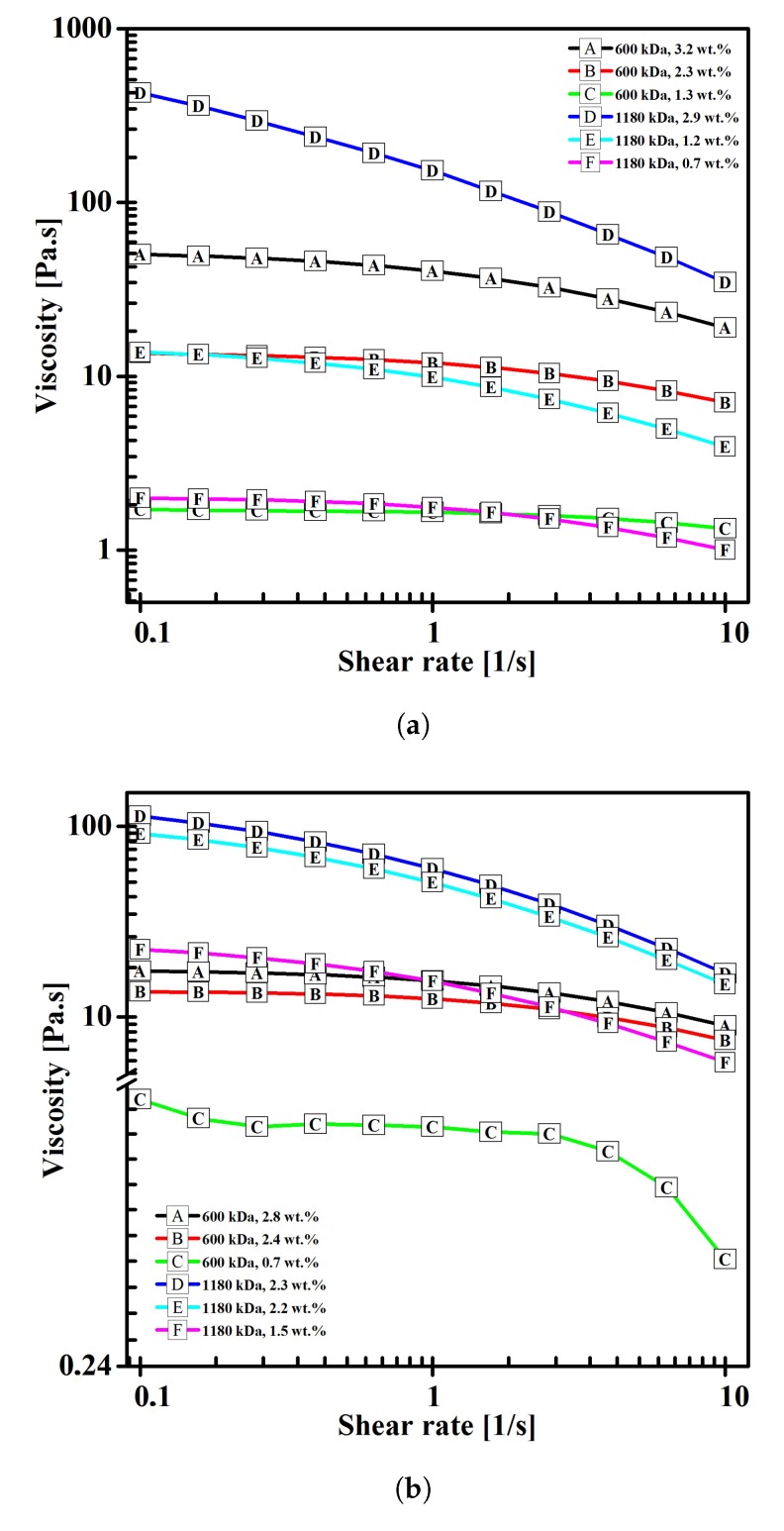
Viscosity of HA solutions in intermediate solvents as a function of shear rate. (**a**) H_2_O:IPA in 10:7 weight ratio solvent mixture. (**b**) H_2_O:EtOH:MeOH in 5:5:1 weight ratio solvent mixture.

The shear viscosity of the solutions decreased with the decrease in both concentration, and $M_{w}$ of the polymer, with the difference being in the range of several orders of magnitude, as is evident from Figure 9.

Viscosity is sometimes considered the determining parameter of spinnability via electrostatic force [18]. The findings of this study contradict such assumption as overly simplified, which is in agreement with Yarin et al. (2001) [46], who claimed that shear viscosity was insignificant in terms of Taylor cone formation. The maximum viscosity of a spinnable solution can differ as much as ten times if different $M_{w}$ of the same polymer are used. We assumed that the determining parameter was polymer chain entanglement, which is affected by the polymer chain conformation in given solvent, the ionic strength of the solution, polymer concentration, and other parameters. Further investigation on this subject is necessary.

Regardless the solvent mixture, spinning voltage decreased with the decrease of concentration (see Table 7 and Table 8), which can possibly be explained by a shift in solution viscoelasticity, causing the critical instability wavelength leading to Taylor cone formation to increase [3], thus consuming less energy and lowering spinning voltage. Since surface tension does not change with concentration, its effect on spinning voltage can be neglected.

No influence of the concentration on the electrospun structure morphology was found, which was the result of little difference in the concentrations of the respective samples. However, the concentration clearly governed the transition between electrospinning and electrospraying, which is explained by polymer chain entanglement according to Shenoy et al. (2005) [39] or by polymer solution elasticity according to Yu et al. (2006) [35]. The mechanism could not clearly be determined from the experiments conducted in the current study. The obtained fibers did not exceed 100 nm in diameter in any of the cases, making them promising in terms of biomedicine. The spherical particle diameter was mostly in the 1 to 0.1 μm range.

**Figure 10:**
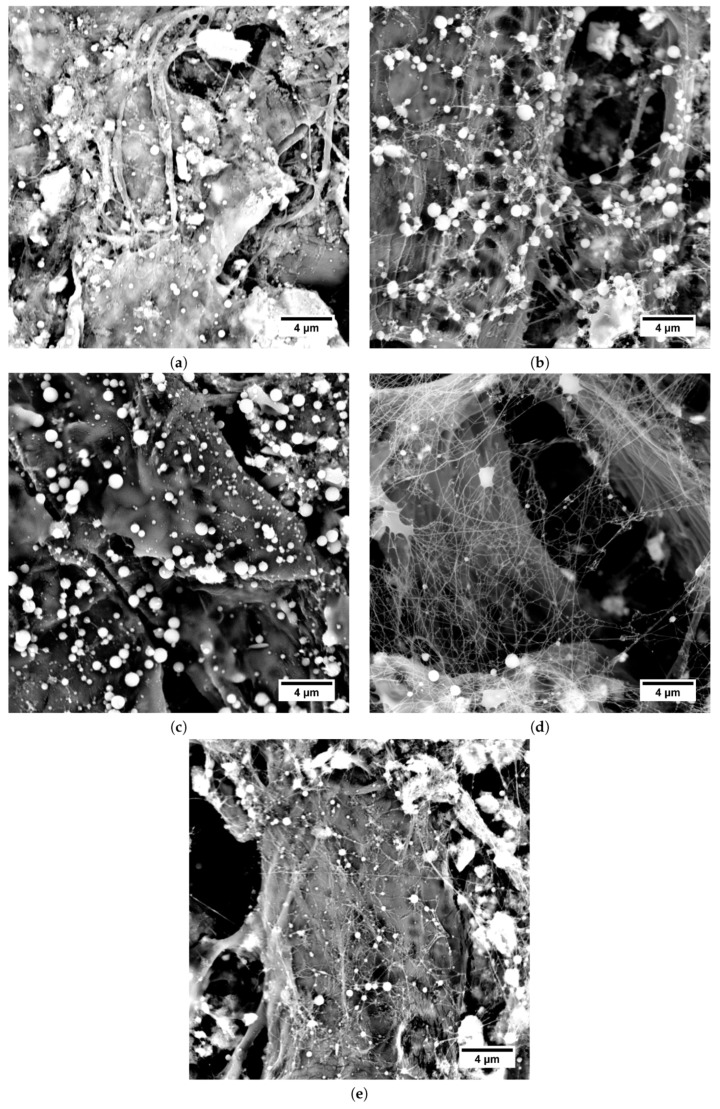
SEM micrographs of electrospun structures obtained from H_2_O:IPA in 10:7 weight ratio solutions. (**a**) 3.2 wt.% HA $M_{w}$ 600 kDa; (**b**) 2.3 wt.% HA $M_{w}$ 600 kDa; (**c**) 1.3 wt.% HA $M_{w}$ 600 kDa; (**d**) 2.9 wt.% HA $M_{w}$ 1180 kDa; (**e**) 1.2 wt.% HA $M_{w}$ 1180 kDa.

**Figure 11:**
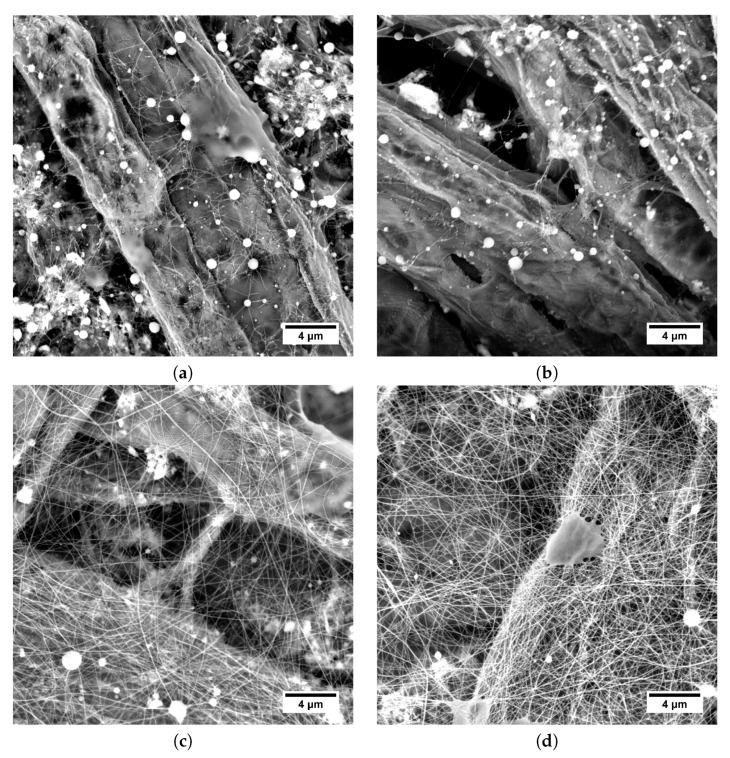
SEM micrographs of electrospun structures obtained from H_2_O:EtOH:MeOH in 5:5:1 weight ratio solutions. (**a**) 2.8 wt.% HA $M_{w}$ 600 kDa; (**b**) 2.4 wt.% HA $M_{w}$ 600 kDa; (**c**) 2.2 wt.% HA $M_{w}$ 1180 kDa; (**d**) 1.5 wt.% HA $M_{w}$ 1180 kDa.

In the case of each solvent mixture, HA 600 kDa showed more tendency to undergo instabilities, which resulted in a combination of electrospinning and electrospraying (Figure 10a–c and Figure 11a–b), whereas HA 1180 kDa was able to provide smooth fibers (Figure 10d and Figure 11c,d). It could be assumed that a higher HA $M_{w}$ was more favorable in terms of electrospinning as a result of the higher elasticity of the solution.

We can also see that instabilities in the form of electrospraying and branching were more frequent for solutions using H_2_O:IPA in 10:7 weight ratio solvent mixture (Figure 10). Solvent mixture H_2_O:EtOH:MeOH in 5:5:1 weight ratio provided higher stability to the electrospinning process. As the conductivity of the solutions using respective solvent mixtures was comparable (see Table 4 and Table 5), the stabilization mechanism was presumably more complex than stabilization by charge density suggested by Reneker and Yarin (2008) [4]. Polymer chain conformation and interaction parameters can be expected to have a significant influence on electrospinning phenomena stabilization, but further investigation is necessary in order to fully understand the process.

Although the conductivity of HA solutions in intermediate solvents was significantly lower than those of aqueous blend solutions with PVA or PEO, smooth fibers were only obtained from a certain HA in intermediate solvent solutions. Our conclusion is that although conductivity does have a positive effect on electrospun jet stabilization, as stated by Reneker and Yarin (2008) [4], the influence of polymer viscoelasticity needs to be taken into account, as suggested by Stepanyan et al. (2014) and Palangetic et al. (2014) [36,37]. In the case of blend-solution electrospinning, the situation is complicated due to the difference in the viscoelastic properties of the polymers, which are simultaneously drawn at high elongation rates. A non-negligible effect of interaction parameters was present as well, because an intermediate solvent has a great influence on the solution behavior in a strong electric field, as was demonstrated in this study, as well as previously [34,39,42].

## 4. Conclusions

Electrospinning of biocompatible and biodegradable polymers is a desirable technique for use in biomedicine and life sciences. Production of HA nanofibers is a challenging task due to the extremely high viscosity and high surface tension of aqueous solutions.

In this study, nanofibers containing HA were obtained by solution electrospinning. Two approaches to the problem were chosen: co-electrospinning of aqueous blend solutions of HA/PVA and HA/PEO, respectively, and use of the intermediate solvent for pure HA solutions electrospinning. The choice of materials was done with regard to potential uses for cell cultivation. To facilitate fiber formation in HA/PVA blend solutions, the addition of BEC was necessary. Both HA/PEO and HA/PVA blend solutions provided bead-on-string structured fibers. As intermediate solvents, H_2_O:IPA in a 10:7 weight ratio and H_2_O:EtOH:MeOH in a 5:5:1 weight ratio were chosen. Both solvent mixtures facilitate the electrospinning of HA of $M_{w}$ 600 and 1180 kDa. Lower $M_{w}$ solutions had higher tendency to form spherical particles. There is clear correlation between the decrease in solution surface tension and the decrease in spinning voltage can be seen in the results, but no significant impact of these parameters on the fiber diameter was found. Variation in electrospun-structure dimensions and morphology was intensely associated with the change in $M_{w}$ of the polymers. It was experimentally demonstrated that shear viscosity cannot be used as a sole determining parameter of solution spinnability, as there are differences as high as ten times the order of magnitude for spinnable solutions that differ only in polymer $M_{w}$.

The best results were achieved with the HA 1180 kDa solution in H_2_O:EtOH:MeOH 5:5:1 at concentrations of 2.2 wt.% and 1.5 wt.%, as these provided smooth fibers. Fiber diameter did not exceed 100 nm for any sample that provided fibers, which makes them promising in terms of tissue engineering.

## Figures and Tables

**Table 1:** Characteristics of Hyaluronan (HA)/Poly(vinyl alcohol) (PVA) blend solution with benzethonium chloride (BEC).

BEC Content [wt.%]	Surface Tension [mN·m^−1^]	Conductivity [μS cm^−1^]
**0.006**	43.7 ± 0.9	1473 ± 7
**0.013**	42.6 ± 0.2	1496 ± 2
**0.033**	41.4 ± 0.6	1513 ± 4
**0.065**	41.1 ± 0.2	1546 ± 4

**Table 2:** Morphology analysis of HA/PVA blend solutions with BEC electrospinning products.

BEC Content [wt.%]	Spinning Voltage [kV]	Product Form	Beads Diameter [μm]	Fibers Diameter [μm]
**0.006**	20.4	Elongated beads	0.1–0.5	/
**0.013**	18.8	Elongated beads	0.3–0.7	/
**0.033**	17.5	Bead-on-string	0.1–0.8	0.03–0.06
**0.065**	15.9	Bead-on-string	0.2–0.6	0.06–0.1

**Table 3:** Characteristics of HA/Polyethylene oxide (PEO) aqueous blend solutions.

PEO $M_{w}$ [kDa]	HA $M_{w}$ [kDa]	Surface Tension [mN·m^−1^]	Conductivity [μS·cm^−1^]
**300**	243	46 ± 2	1284 ± 8
	370	49.3 ± 0.2	1255 ± 7
	600	49 ± 2	1241 ± 6
**600**	243	51 ± 2	1312 ± 4
	370	52 ± 2	1258 ± 3
	600	54 ± 1	1213 ± 3

**Table 4:** Characteristics of HA solutions in H_2_O:Isopropanol (IPA) in 10:7 weight ratio solvent mixtures.

$M_{w}$ [kDa]	Concentration [wt.%]	Surface Tension [mN·mm^−1^]	Conductivity [μS·cm^−1^]
**600**	3.2	27 ± 1	854 ± 2
	2.3	25 ± 0.5	568 ± 1
	1.3	26.2 ± 0.3	465 ± 1
**1180**	2.9	24 ± 3	682 ± 2
	1.2	28 ± 2	386 ± 2
	1.0	27.1 ± 0.5	343 ± 2

**Table 5:** Characteristics of HA solutions in H_2_O:Ethanol (EtOH):Methanol (MeOH) in 5:5:1 weight ratio solvent mixtures.

$M_{w}$ [kDa]	Concentration [wt.%]	Surface Tension [mN·m^−1^]	Conductivity [μS·cm^−1^]
**600**	2.8	32.3 ± 0.9	740 ± 8
	2.4	30.5 ± 0.5	748 ± 1
	0.7	30.2 ± 0.8	249 ± 1
**1180**	2.3	27.4 ± 0.8	704 ± 1
	2.2	28 ± 2	616 ± 1
	1.5	27.6 ± 0.9	459 ± 1

**Table 6:** Morphology analysis of HA/PEO blend-solution electrospinning products.

PEO $M_{w}$	HA $M_{w}$	Spinning Voltage [kV]	Product Form	Beads Diameter [μm]	Fibers Diameter [μm]
300	243	18.2	Bead-on-string	0.25–0.5	0.02–0.05
	370	18.2	Bead-on-string	0.3–0.5	0.03–0.07
	600	18.2	Bead-on-string	0.2–0.6	0.04–0.1
600	243	24.5	Bead-on-string	0.2–0.7	0.05–0.1
	370	24.5	Bead-on-string	0.3–0.6	0.05–0.1
	600	24.5	Bead-on-string	0.3–0.6	0.03–0.09

**Table 7:** Morphology analysis of H_2_O:IPA in 10:7 weight ratio HA solutions electrospinning products.

HA $M_{w}$ [kDa]	Concentration [wt.%]	Spinning Voltage [kV]	Product Form	Particles Diameter [μm]	Fibers Diameter [μm]
**600**	3.2	20.5	Spherical Particles; Fibers	0.3–0.6	0.04–0.1
	2.3	20.0	Spherical Particles; Fibers	0.3–1.0	0.06–0.1
	1.3	16.2	Spherical Particles; Fibers	0.4–1.2	0.05–0.1
**1180**	2.9	24.0	Fibers	/	0.05–0.09
	1.2	16.3	Spherical Particles; Fibers	0.3–0.8	0.06–0.1
	1.0	19.0	Spherical Particles	0.7–1.1	/

**Table 8:** Morphology analysis of H_2_O:EtOH:MeOH in 5:5:1 weight ratio HA solutions electrospinning products.

HA $M_{w}$ [kDa]	Concentration [wt.%]	Spinning Voltage [kV]	Product Form	Particles Diameter [μm]	Fibers Diameter [μm]
**600**	2.8	19.1	Spherical Particles; Fibers	0.4–1.0	0.05–0.07
	2.4	16.5	Spherical Particles; Fibers	0.2–0.8	0.05–0.07
	0.7	14.9	Spherical Particles	0.3–1.2	/
**1180**	2.3	29.2	/	/	/
	2.2	22.9	Fibers	/	0.05–0.08
	1.5	19.2	Fibers	/	0.05–0.1

## Abbreviations

The following abbreviations are used in this manuscript:

HA	Hyaluronan
DC	Direct current
PVA	Poly(vinyl alcohol)
PEO	Polyethylene oxide
$M_{w}$	Average molecular weight
PEG	Polyethylene glycol
EtOH	Ethanol
IPA	Isopropanol
MeOH	Methanol
DEMI	Demineralized
DMSO	Dimethyl sulfoxide
FITC	Fluorescein isothyocyanite isomer
BEC	Benzethonium chloride
SEM	Scanning Electron Microscope/Scanning Electron Microscopy
